# Cord Blood Ischemia-Modified Albumin Levels in Normal and Intrauterine Growth Restricted Pregnancies

**DOI:** 10.1155/2008/523081

**Published:** 2008-05-07

**Authors:** Nicoletta Iacovidou, Despina D. Briana, Maria Boutsikou, Sophia Liosi, Stavroula Baka, Theodora Boutsikou, Demetrios Hassiakos, Ariadne Malamitsi-Puchner

**Affiliations:** Neonatal Division, 2nd Department of Obstetrics and Gynecology, Athens University Medical School, 10682 Athens, Greece

## Abstract

Ischemia-modified albumin (IMA) is a sensitive biomarker of cardiac ischemia. Intrauterine growth restriction (IUGR) may imply fetal hypoxia, resulting in blood flow centralization in favour of vital organs (brain, heart, adrenals—“brain sparing effect”). Based on the latter, we hypothesized that cord blood IMA levels should not differ between IUGR and appropriate-for-gestational-age (AGA) full-term pregnancies. IMA was measured in blood samples from doubly-clamped umbilical cords of 110 AGA and 57 asymmetric IUGR pregnancies. No significant differences in IMA levels were documented between AGA and IUGR groups. IMA levels were elevated in cases of elective cesarean section (*P* = .035), and offspring of multigravidas (*P* = .021). In conclusion, “brain sparing effect” is possibly responsible for the lack of differences in cord blood IMA levels at term, between IUGR and AGA groups. Furthermore, higher oxidative stress could account for the elevated IMA levels in cases of elective cesarean section, and offspring of multigravidas.

## 1. INTRODUCTION

Ischemia-modified albumin (IMA) is a relatively new biomarker in the identification of myocardial ischemia in advance or in the absence of myocardial necrosis [1]. Since IMA can be detected before troponin (a well-known marker of necrosis) and has a high sensitivity (82%) compared to traditional diagnostic tools (ECG-45%, troponin-20%), it is valuable for the diagnosis of myocardial ischemia in patients presenting with chest pain at the emergency department [1–3]. It is well known that IMA rises within minutes from the onset of the ischemic event and remains elevated for several hours after cessation of ischemia [2, 3].

The pathophysiological events of ischemia, including hypoxia, acidosis, and free radical generation, result in a conformational change of the N-terminus of albumin, leading to a reduction in its binding with the transition metals copper, nickel, and cobalt [4, 5]. The resulting molecule, IMA and its reduced binding of cobalt, has been exploited to produce a rapid automated test, the albumin cobalt binding assay [6]. Intrauterine growth restriction (IUGR) caused by chronic malnutrition and hypoxia, (consequent to deficient placental transport of nutrients and oxygen—asymmetrical pattern of IUGR)—is characterized by blood flow redistribution to vital organs (brain, myocardium, and adrenal glands), while other organs are deprived from sufficient blood flow [7–9]. This phenomenon called “the brain sparing effect” is usually accompanied by oligohydramnios [10, 11].

Taking into account “the brain sparing effect,” this study was based on the hypothesis that cord blood IMA levels should not differ between IUGR and appropriate for gestational age (AGA) full-term pregnancies. Therefore, we aimed to investigate cord blood IMA levels in IUGR and AGA pregnancies at birth and correlate determined levels with gestational age, gender, and mode of delivery.

## 2. SUBJECTS AND METHODS

The Ethics Committee of our teaching hospital approved the study protocol. All included mothers provided signed informed consent before recruitment. In the time period from January 2006 to January 2007, one hundred and sixty seven parturients giving consecutively birth either to AGA (*n* = 110) or IUGR (*n* = 57), birth weight below the 3rd customized centile) full-term singleton infants were included in the study. Deliveries were performed either vaginally or by elective caesarean section. The gestation related optimal weight (GROW) computer-generated program [12, 13] was used to calculate the customized centile for each pregnancy, taking into consideration significant determinants of birth weight, such as maternal height and booking weight, ethnic group, parity, gestational age, and gender [12].

Causes of IUGR were identified in each one of our 57 IUGR cases. Thus, 13 mothers presented with pre-eclampsia, 20 with pregnancy-induced hypertension, 3 with severe type-I diabetes mellitus, while 21 were heavy smokers during the whole duration of pregnancy. Amniotic fluid was diminished in all IUGR cases. For the evaluation of the amniotic fluid, the largest fluid column on the vertical plane was assessed and was defined as diminished, if < 2 cm. Furthermore, placental weight was reduced [14] ranging from 230 to 420 g.

In contrast, in the AGA group, mothers were healthy and were either nonsmokers or had abstained from smoking during pregnancy. Moreover, placentas were normal in appearance and weight.

All neonates had no symptoms of intrauterine infection or stigmata of genetic syndromes. One- and five-minute Apgar scores were ≥8 and umbilical cord pH values were ≥7.25 in all IUGR cases and AGA controls. The demographic data of participating infants and mothers are shown in Table 1.

Blood was collected at birth, from the doubly-clamped umbilical cord (UC), reflecting the fetal state. Pyrogen-free tubes were used and samples were immediately centrifuged after clotting. The supernatant serum was stored at −80°C until assay.

IMA was measured with the albumin cobalt binding (ACB) test (inverness medical professional diagnostics, Bedford, UK). A cobalt solution is first added to serum, followed by addition of dithiothreitol (DTT). DTT reacts with the cobalt to form a colour that is measured by absorbance read spectrophotometrically. In serum of normal patients, cobalt binds to the N-terminus of albumin, which leaves little cobalt to react with DTT, producing a lighter colour. Conversely, in serum of ischaemic patients, cobalt does not bind to the N-terminus of IMA, which leaves more free cobalt to react with DTT, forming a darker colour. The ACB test is configured to run on Roche Cobas Integra 800 instrument. The minimum detectable concentrations, intra- and inter-assay coefficients of variation were 28 U/ml, 1.7% and 5.7%, respectively.

## 3. STATISTICAL ANALYSIS

Data regarding IMA were normally distributed (Kolmogorov Smirnov test). Linear regression analysis was used to examine the effect of gender, mode of delivery, parity, birth-weight, and gestational age on IMA levels. Student's *t*-test was used to detect differences in continuous variables (IMA, birthweight, gestational age) between IUGR and AGA groups. Mann Whitney U test was used to detect differences in customized centiles between groups, since data regarding customized centiles were not normally distributed. Pearson's chi square test was used to detect differences concerning gender, mode of delivery and parity between groups. Spearman's or Pearson's correlation coefficient were used to detect any positive or negative correlations. *P* < .05 was considered statistically significant. The power calculation of the study was 85%.

## 4. RESULTS

Doppler studies were performed in the IUGR group every 10–15 days, starting from the 32nd gestational week. During each Doppler velocimetry evaluation study, three consecutive measurements of the pulsatility index (PI) of the studied vessel were done and the mean value was recorded. Concerning uterine and umbilical arteries [15, 16], mean PI values were progressively found to be in the upper physiological limits for the corresponding gestational age in 38 cases (ranging between the 90th and the 95th percentile), while in the remaining 19 cases PI values showed increased impedance to flow, being above the 95th percentile for gestational age. Regarding middle cerebral arteries [17], Doppler studies showed resistance to be in the lower physiological limits for gestational age, indicating the initiation of blood flow redistribution process. Determined mean [95% confidence intervals (CI)] values of cord blood IMA levels were in the AGA group 112.44 (109.95–114.92) and in the IUGR group 115.54 (111.97–119.12). No significant differences in cord blood IMA levels were found between IUGR cases and AGA controls. In both groups, cord blood IMA levels were significantly elevated in cases of caesarean section (by 4.676 IU/mL on average) compared to cases of vaginal delivery (*b* = 4.676, CI 95%: 0.328–9.025, *P* = .035). Additionally, cord blood IMA levels were significantly increased (by 5.012 IU/mL on average) in cases of multigravidas compared to primigravidas (*b* = 5.072, CI 95%: 0.768–9.256, *P* = .021). Finally, cord blood IMA levels did not depend on gestational age or gender.

## 5. DISCUSSION

It has been recently reported that IMA is a highly sensitive biomarker, reflecting the myocardial ischemic condition prior to progression to myocardial necrosis [18–20]. In this respect, IMA has been reported to increase after percutaneous coronary interventions and in acute coronary syndromes [1, 21]. In this setting, elevated IMA levels may result from increased oxidative stress, caused by ischemia reperfusion injury or other mechanisms linked to primary reduction of coronary blood flow or muscle damage [1, 21–23].

Although there is a growing body of evidence regarding IMA in the area of monitoring acute coronary syndromes in adults [1, 18–23], data concerning the perinatal period are sparse [24, 25]. In this respect, higher cord blood IMA values were documented in complicated deliveries, indicating fetal distress and/or hypoxia [24]. Furthermore, elevated maternal serum IMA levels have been recently demonstrated in early normal pregnancy, supporting the hypothesis that normal trophoblast development is associated with a hypoxic intrauterine environment [25].

In line with our hypothesis, the results of the present study indicate a lack of significant differences in cord blood IMA levels between nondistressed IUGR cases and AGA controls, suggesting no evidence of myocardial damage in the former, possibly due to sparing of vital organs (e.g., brain, heart) [7–9]. Similarly, previous studies of ours reported that circulating levels of neurotrophins, which are important for pre- and postnatal brain development, as well as of cardiac troponin-I, which is a highly specific indicator of myocardial damage, did not differ between asymmetric nondistressed IUGR and AGA groups [26, 27].

On the other hand, contrary to our expectations, cord blood IMA levels were significantly elevated in cases of elective caesarean section compared to cases of vaginal delivery. In the literature, no evidence exists whether the fetus is subject to oxidative stress during labour [28]. Several studies have shown that umbilical arterial blood of newborns delivered vaginally or after emergent caesarean section contains higher levels of oxidation products than the blood of newborns delivered by elective caesarean section [29–32]. However, other reports suggest that oxidative stress in the fetal circulation does not depend on the mode of delivery [28]. In fact, a recent study even found that the “reservoir” of antioxidants in the fetal red blood cell compartment is higher in infants delivered vaginally than in those delivered by elective caesarean section [33]. It should be noted that all neonates included in our study were born healthy, as evaluated by Apgar scores and pH values.

Moreover, in our cohort, cord blood IMA levels were found to depend on parity, being significantly elevated in offspring of multigravidas. Relatively, epidemiological studies have demonstrated a consistent relationship between the number of pregnancies and the subsequent development of cardiovascular disease, due to an increase in oxidative damage during late pregnancy [34, 35]. The association, found between cord blood IMA levels at term and parity in the present study, may have important implications in the development of atherosclerosis and long-term cardiovascular health of women of high parity [34, 35].

In conclusion, cord blood IMA levels at term do not differ between IUGR cases and AGA controls, possibly due to the sparing of vital organs, like the heart. Furthermore, higher oxidative stress may account for the elevated cord blood IMA levels in cases of elective caesarean section, as well as in the offspring of multigravidas. Oxidative stress indicated by elevated IMA levels in women of high parity may have important implications in their long-term cardiovascular health and warrants further investigation.

## Figures and Tables

**Table 1:** Demographic data of appropriate for gestational age (AGA) and intrauterine growth restricted (IUGR) pregnancies.

	AGA Mean ± SD (Median)	IUGR Mean ± SD (Median)	*P*
Birth-weight (g)	3195 ± 298.5 (3200)	2595 ± 264.5 (2630)	< 0.001
Customized Centiles	40.1 ± 23 (35)	5.7 ± 5.2 (5)	< 0.001
Gestational age (weeks)	39.0 ± 1.0 (39.1)	38.4 ± 1.4 (38.3)	NS
Gender	—	—	NS
Male	66 (60)	27 (47.4)	—
Female	44 (40)	30 (52.6)	—
Mode of delivery	—	—	0.022
Vaginal	82 (74.5)	32 (56.1)	—
Elective Caesarean section	28 (25.5)	25 (43.9)	—
Parity	—	—	NS
First	79 (71.8)	37 (64.9)	—
Other	31 (28.2)	20 (35.1)	—

NS: nonsignificant.
